# A whole-task brain model of associative recognition that accounts for human behavior and neuroimaging data

**DOI:** 10.1371/journal.pcbi.1011427

**Published:** 2023-09-08

**Authors:** Jelmer P. Borst, Sean Aubin, Terrence C. Stewart

**Affiliations:** 1 Bernoulli Institute, University of Groningen; Groningen, The Netherlands; 2 Centre for Theoretical Neuroscience, University of Waterloo; Waterloo, Ontario, Canada; 3 National Research Council Canada, University of Waterloo Collaboration Centre; Waterloo, Ontario, Canada; Brown University, UNITED STATES

## Abstract

Brain models typically focus either on low-level biological detail or on qualitative behavioral effects. In contrast, we present a biologically-plausible spiking-neuron model of associative learning and recognition that accounts for both human behavior and low-level brain activity across the whole task. Based on cognitive theories and insights from machine-learning analyses of M/EEG data, the model proceeds through five processing stages: stimulus encoding, familiarity judgement, associative retrieval, decision making, and motor response. The results matched human response times and source-localized MEG data in occipital, temporal, prefrontal, and precentral brain regions; as well as a classic fMRI effect in prefrontal cortex. This required two main conceptual advances: a basal-ganglia-thalamus action-selection system that relies on brief thalamic pulses to change the functional connectivity of the cortex, and a new unsupervised learning rule that causes very strong pattern separation in the hippocampus. The resulting model shows how low-level brain activity can result in goal-directed cognitive behavior in humans.

## Author summary

Despite large research enterprises in the past decade, it is still unknown how our cognitive system is implemented by the immense complexity of the neurons in our brains. Here, we present a large-scale computational model of the human brain that explains how low-level neural activity can lead to functional, goal-directed behavior. Previous models typically either focused on low-level biological detail or on part-task, qualitative behavioral effects. In contrast, the current model is biologically plausible through the use of spiking neurons, and accounts both for human behavior and neuroimaging data across a whole task. The model’s mechanisms are based on detailed machine-learning analyses of neuroimaging data, as well as on cognitive theories of associative recognition.

## Introduction

Despite large research initiatives (e.g., [1]), it is still an open question how our cognitive system is implemented by the immense complexity of the interconnected neurons of the human brain, that is, how low-level neural activity can lead to functional, goal-directed behavior. Traditionally, neural network models with simplified neurons have addressed this question. These models typically focused on only part of a task–for example, only on memory processing or only on perception–and on qualitative behavioral effects (e.g., [2]). More recently, several large-scale biologically-plausible models of the brain have been presented (for reviews, see [3,4]). While these models simulated low-level neural anatomy in tremendous detail, they omitted the connection to functional behavior.

In 2012, Eliasmith and colleagues presented a 2.5-million-neuron brain model that combined biological plausibility through the use of spiking neurons with the effective performance of eight different tasks [5]. This seminal model indicated how behavior could theoretically follow from low-level neural activity. However, its evaluation was limited to qualitative comparisons with human behavior and several local neural effects, which did not fully account for the necessarily immense complexity of the model.

In this paper, we go a step further, and present a brain model consisting of 810k spiking neurons that performs associative learning and recognition. Following a recent suggestion by Kriegeskorte and Douglas [6], this model combines insights from cognitive science with machine-learning analyses of encephalography (EEG) and magnetoencephalography (MEG) data. To evaluate the model, we compared its results not only quantitatively to human behavior, but also qualitatively to ongoing source-localized MEG activity across five brain regions and to a classic fMRI effect.

The specific task that the brain model performed was *associative recognition*: judging whether two items were experienced together in the past. Associative recognition involves a variety of cognitive processes that are present in many tasks [7], ensuring that the simulated cognitive mechanisms are applicable more generally. In addition, there are extensive experimental results [8,9] and converging high-level models and theories on the cognitive mechanisms underlying associative recognition [10–12], providing a strong foundation for the model.

## Results

### Theoretical model based on EEG and MEG data

The brain model (available at https://doi.org/10.5281/zenodo.7988965) was used to simulate data collected previously across an EEG [8] and an MEG experiment [9]. In both studies, participants studied 32 word pairs in a training phase. In a subsequent test phase, they were asked to distinguish between three different *probe types*: targets (trained pairs, *yes* response), re-paired foils (rearranged target pairs, *no* response), and new foils (completely new words, *no* response). To be able to distinguish between targets and re-paired foils, participants did not only need to memorize the words themselves, but also the associations between the words in a pair–making the task *associative* recognition. In addition to probe type, three other factors were manipulated: word length (long or short), response hand (left or right), and fan (one or two; indicating whether both words in a pair appeared uniquely in one pair or in two different pairs). Fan is well known to have strong effects on reaction times and error rates, with higher fan leading to more mistakes and longer response times [13,14]. In addition, higher fan pairs cause higher levels of BOLD activity in the dorsolateral prefrontal cortex [15–17]. Details of the experiments can be found in the Materials and Methods.

**Fig 1:**
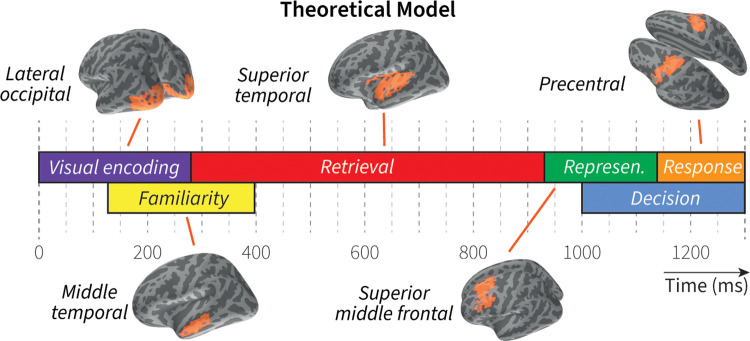
Theoretical model of associative recognition. Boxes indicate processing stages based on machine-learning analyses of EEG data; associated regions-of-interest are based on MEG evidence. Represen. = representation.

As basis for the brain model, we used a theoretical model of associative recognition that we developed previously [8,12,18]. This theoretical model was based on the results of a machine-learning method that can discover cognitive processing stages in EEG data, and combined elements of three existing cognitive theories of associative recognition: signal-detection theory [10], dual-process theories [11], and the cognitive architecture ACT-R [13]. Fig 1 shows the stages of the theoretical model. It starts by visually encoding the words presented on the screen, during which a familiarity stage starts. If the encoded words are unfamiliar–indicative of a *new foil*–a *no* response is given. If the encoded words are familiar, a retrieval stage commences instead, in which the most similar word pair to the encoded pair is retrieved from memory. The retrieved pair is represented in a temporary store, and compared to the encoded stimuli to decide between *targets* (the encoded pair is the same as the retrieved pair) and *re-paired foils* (the encoded pair is different from the retrieved pair); a so-called recall-to-reject process [19].

To localize these cognitive mechanisms in the brain, we performed a follow-up MEG study [9]. Using participants’ individual structural MRIs, we projected the MEG sensor data on 3D reconstructions of their cortical surfaces (using cortically constrained minimum norm estimates, MNE; [20]). To identify time periods and cortical sources associated with the cognitive mechanisms involved in associative recognition (Fig 1), two analysis methods were used [9]. First, nonparametric cluster-based permutation tests [21] were applied to identify temporo-spatial clusters of cortical sources associated with the experimental conditions, which, in turn were associated with specific cognitive mechanisms. Second, multivariate ridge regression classification [22] was applied across the whole brain and separately in 68 cortical regions (Desikan-Killiany atlas [23]) to improve the temporal and spatial definition. By integrating the results of these analyses, one main cortical region for each cognitive mechanism–except for the Decision process–was identified, well-defined in time (Fig 8 in the original paper [9]).

To evaluate the brain model, we created five regions-of-interest (ROIs) based on these results, which should be indicative of the different cognitive mechanisms. For each mechanism, we took the intersection of significant sources from the non-parametric permutation test for a specific experimental contrast (see the original paper [9] for argumentation on contrast selection) and important cortical regions as identified by the classification analysis. For example, for right-handed motor actions (cognitive mechanism), we intersected significant sources indicated by the effect of response hand (left vs. right, permutation test) with the left pre-central cortical area (classification analysis). The resulting ROI is visualized in Fig 1. S1 Table lists all the used contrasts and cortical regions, as well as properties of the ROIs.

### Brain Model—Architecture

**Fig 2:**
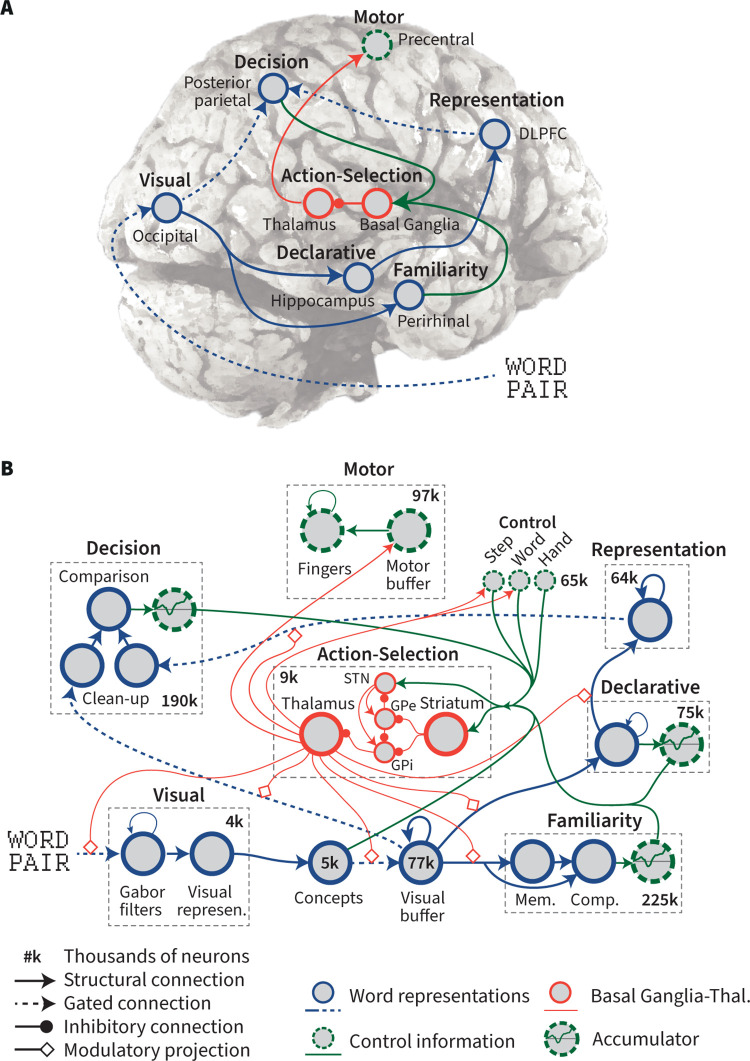
Architecture of the brain model. A) high-level overview of the seven main modules with their connections and rough localization on the brain, B) detailed functional architecture including neuron counts. DLPFC = dorsolateral prefrontal cortex; STN = subthalamic nucleus; GPe = globus pallidus externus, GPi = globus pallidus internus; Mem. = memory; Comp. = comparison.

The brain model implements the cognitive mechanisms of the theoretical model in Fig 1, and carries out the experiment described above. Fig 2A shows the high-level architecture of the brain model, which consists of seven main modules (S2 Table). Each module was linked to a specific brain region, which represents the main processing hub for the associated cognitive function. As a result, activity in this brain region–as covered by the ROIs–should be indicative of the cognitive function. Fig 2B shows the detailed functional architecture of the modules. We describe these modules here in terms of their functional behavior, but it must be stressed that the underlying neural simulation consists entirely of spiking neurons with synaptic connections between them. As we validated the model using continuous neural data, it was required to do the exact same task as human participants, and therefore received the same stimuli as input, and responded with a simulated key press.

### Nengo framework

The model was implemented in the Nengo framework, a Python library aimed at building large-scale spiking-neuron models that link low-level neural activity to cognitive processing and overt behavior [24–26]. In Nengo, information is represented by real-valued vectors, which can be encoded and decoded from the collective spiking activity of neural populations [26]. By default, leaky-integrate-and-fire (LIF) neurons are used, which encode information through non-linear tuning curves that characterize their response to the incoming signal.

To represent symbols–for example, the words in the associative recognition experiments–we used *semantic pointers* [26,27]. Semantic pointers are compressed representations that carry partial semantic content, implemented as high-dimensional vectors in Nengo [5,26]. We used 512-dimensional semantic pointers to represent the words and word pairs. The direction of a semantic pointer/vector represents its content, while its length represents the clarity of the representation. For example, the words ‘brain’ and ‘neuron’ would be represented by 512D vectors with a different direction (in the current model these directions are determined randomly, however, in theory related words should have similar directions [27]). Perfectly represented vectors have a length of 1. If a vector is shorter, it is more susceptible to noise and can therefore be confused with other vectors.

Vectors–and thus semantic pointers–can be communicated and transformed through neural connections. In contrast to most neural frameworks, connection weights do not have to be learned in Nengo, but can be pre-calculated to approximate a given function, allowing for the quick development of large-scale models (see Methods for details). To mimic neural plasticity, connection weights can also be learned and adapted through several biologically-plausible learning rules [28–30]. Connection weights are fully based on functions that communicate and perhaps transform vectors, which has the effect that neural activity itself is not directly transferred between populations.

### Action selection

In agreement with the literature [31–34], Nengo employs a detailed model of the basal ganglia-thalamus complex as an action-selection system to coordinate the flow of information between cortical regions [35]. Following current theories, the striatum monitors the state of all cortical areas. When this state matches a predefined state, an action is selected and executed through the thalamus, which can change the functional connectivity between different brain regions. By changing the connectivity–for example, opening a gate between visual working memory and the declarative memory system–a new stage of processing is started (see Fig 2B for incoming and outgoing connections of the basal ganglia-thalamus system).

In the default Nengo implementation and similar models [31,33–35], such a connectivity change is maintained for as long as the state of the cortex matches the predefined striatal state by continuous input from the thalamus. Based on theories of EEG generation [12,36–38], we adapted this to a system that only uses *brief* thalamic pulses to adjust cortical connections. It was recently shown that such brief pulses can lead to sustained periods of adjusted effective connectivity [39]. In these periods the brain continues to process information, until a new cortical state is reached that matches another predefined striatal state, which changes the functional connectivity again. Not only is this system more in line with the neuroscientific literature, but it also resulted in a close match of the source-localized MEG data.

### Memory system

Before the brain model performed the associative recognition task, we pre-trained the model’s memory system with the word pairs that participants saw in training, following the same presentation rates as experienced by the human participants. Importantly, fan-2 pairs were presented about twice as often as fan-1 pairs.

**Fig 3:**
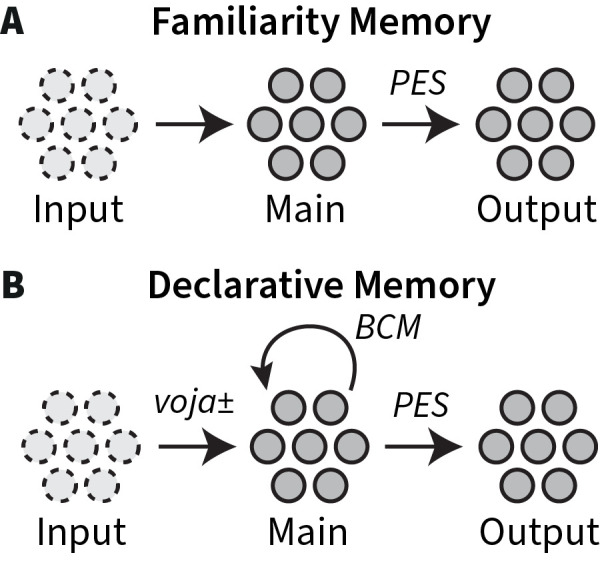
Memory systems. Both familiarity memory and declarative memory receive input from other parts of the model. (A) Familiarity memory learns to associate output with a certain input through supervised PES learning. (B) In addition, declarative memory creates very sparse representations in the main module through unsupervised Voja± learning, and uses recurrent strengthening to bind patterns together through unsupervised BCM learning.

To faithfully simulate familiarity judgement and memory retrieval, two cognitive processes that play a major role in associative recognition [9,10,40,41], our memory system implemented a simplified version of Norman and O’Reilly’s Complimentary-Learning-Systems (CLS) framework [2,42]. This framework consists of two components: given a certain input, the familiarity system (perirhinal cortex) quickly judges the similarity to existing memory items, and the declarative memory system (hippocampus) retrieves detailed information. Fig 3 shows our implementation of this framework. Both memory systems consist of a main population and an output layer, and receive input from other parts of the model. To learn to represent the presented information, the locally-supervised Prescribed Error Sensitivity (PES) learning rule [43] was applied to the connection between the main population and the output layer of both systems.

To match the stronger pattern separation in the declarative memory system of the CLS framework–and in the human hippocampus [44]–we added Hebbian learning to the incoming connections of the declarative memory system, making the neurons more selective to previously presented input vectors. To this end, we developed a new learning rule: Voja±. Voja± is an adjusted version of the existing vector-Oja learning rule called Voja [45], which, in turn, is based on the well-known Hebbian Oja rule [46].

Although the standard Voja rule already leads to pattern separation, thereby avoiding catastrophic forgetting and yielding robust learning [29], the resulting neural activity did not match the measured data. In the Voja± learning rule, we therefore added a threshold term (see Materials and Methods). Due to this threshold term, only relatively strongly responding neurons are further attracted to the input vectors, while weaker responding neurons are repelled. This is similar to sharpening effects through inhibitory competition [2,47], but with this new mechanism no inhibitory feedback connections are required. The result is very strong pattern separation, which is typically associated with the hippocampus [44].

Finally, a recurrent all-to-all connection was added to the main population of the declarative memory system to bind the elements of an activated pattern together. The weights of this connection were learned using the unsupervised Hebbian BCM learning rule [48] with a weight limit.

### Brain Model—Process

**Fig 4:**
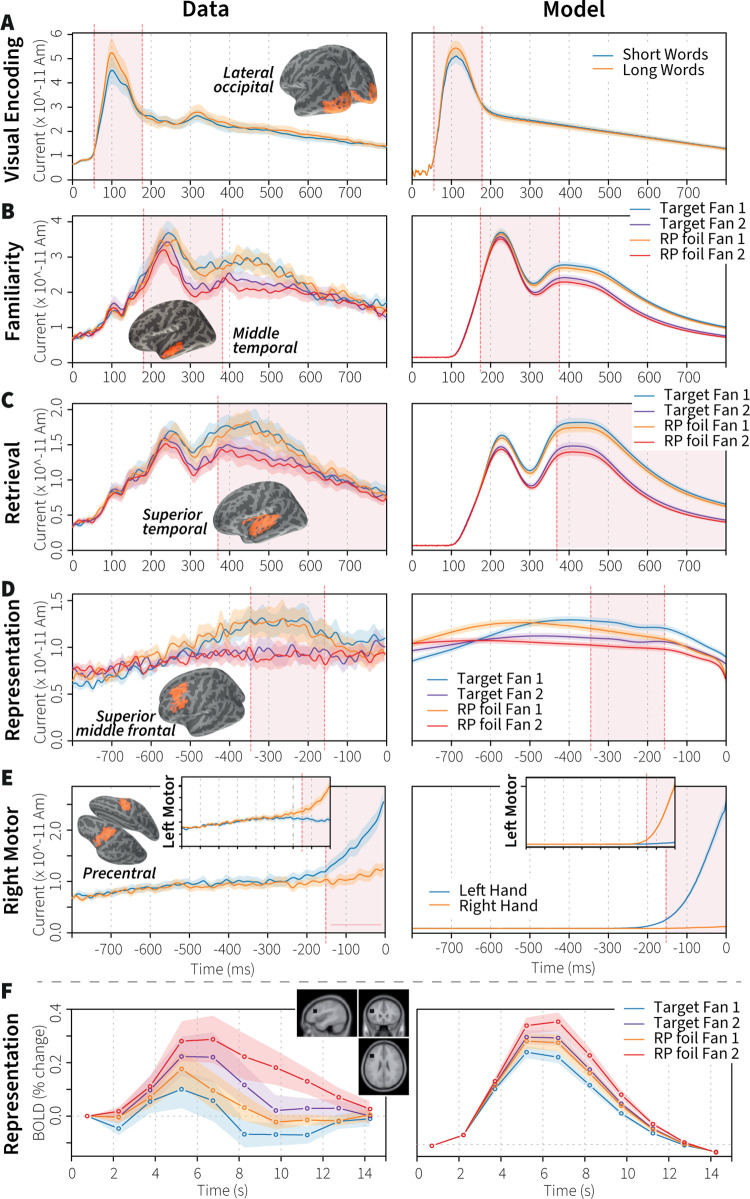
**MEG data (A-E), fMRI data (F), and model fit**. Shaded areas indicate time windows associated with the respective processes. RP foil = re-paired foil. A-C show stimulus-locked data, D-E response-locked. Because the hippocampus and perirhinal cortex are adjacent, both regions contribute to ‘familiarity’ and ‘retrieval’ data. Model fits for these regions are mixtures of the familiarity and memory populations. S2 Fig shows pure familiarity and declarative memory activity.

Each trial of the experiment started with the presentation of a word pair (S1 Fig). The first word was registered about 30 ms later by the occipital cortex [12], leading to a peak in activity that was more pronounced for long than for short words in the human data (Fig 4A, left). The words were presented to the model as images, and the model simulated this stage of visual processing by attending both words in turn, parsing the images of the words with neurons that use Gabor filters as tuning curves [26]. Longer words–more black pixels–yielded a higher neural response (Fig 4A, right), in line with theories of this effect [49]. A weak recurrent connection caused the long tail of the activity. In the remainder of the simulated visual hierarchy [5,26,30], the parsed images were mapped onto lower dimensional visual representations, which in turn drove neurons in a concept population to represent two semantic pointers for the words. These semantic pointers were maintained in a visual buffer for the remainder of the trial (Fig 2B).

Following the theoretical model discussed above, the familiarity of the words was judged next. This process has been located to the perirhinal cortex [9,11], but is projected onto the middle temporal cortex with MEG source localization [9,50]. The peak in activity lies between 180 and 320 ms (Fig 4B). Following signal-detection theories [10], the model compared the encoded words to all words in the familiarity memory population simultaneously and accumulated evidence of their similarity.

**Fig 5:**
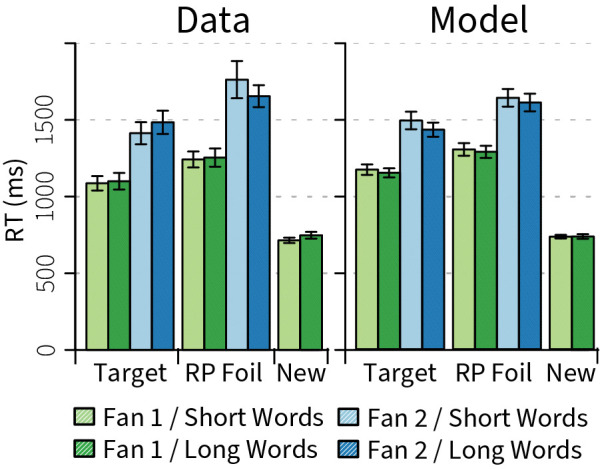
Response time data and model fit. Error bars indicate standard errors of the mean. RP foil = re-paired foil.

To this end, the word concepts were used as input to the familiarity memory, activating all related memories. To calculate a familiarity index, the input was compared to the output of the familiarity memory using an additional neural population. The resulting one-dimensional scalar was compared to a threshold; depending on whether it was above or below the threshold, a positive or negative signal was transmitted to an accumulator. In accordance with known neural evidence accumulation systems [51–53], the accumulator collected evidence for or against a familiar decision. If the evidence exceeded a positive threshold, the model continued to the retrieval stage. If it exceeded a negative threshold–indicative of a new foil–it directly issued a ‘no’ response, explaining the fast responses to new foils (Fig 5).

In case the words were judged to be familiar, the model retrieved the word pair most similar to the presented pair from declarative memory and stored it in the prefrontal representation (Fig 2). On target trials, the presented pair was typically retrieved, whereas on re-paired foil trials, a pair containing one of the presented words was retrieved–as there is no trace in memory containing the presented pair itself. The duration of the retrieval depended on the quality of the retrieved information. The length of the vector in the output layer of the declarative memory system–indicative of the clarity of the representation–was used as input for an accumulator. When this accumulator reached a certain threshold, the retrieval was deemed finished. For fan-2 pairs, this retrieval process took longer than for fan-1 pairs, because fan-2 pairs were encoded less well in memory as they are hard to distinguish from related fan-2 pairs.

Fig 4C shows data from the superior temporal region, reflecting memory-related activity in the hippocampus, and Fig 4D from representation activity in the dorsolateral prefrontal cortex. In these regions, fan-1 pairs caused higher activation levels than fan-2 pairs. This might seem surprising [9], given that fan-2 words are related to more items in memory, have been seen more than twice as often in training, and because a classic fMRI effect shows the opposite effect (Fig 4F). To simulate this, we used the new Voja± learning rule, which causes neurons that already respond relatively strongly to a certain input to specialize further, while all other neurons are repelled from this specific input (see above and Materials and Methods). Because more neurons are repelled than attracted, stimuli that were seen more often during training–fan-2 pairs–yield a weaker neural response after learning (Fig 4C), which carries over to the prefrontal representation (Fig 4D).

According to most theories of associative recognition, decisions between *targets* and *re-paired foils* are made through a recall-to-reject mechanism [2,11–13,19]. After the retrieval was finished, the model therefore compared the encoded pair in the visual buffer to the retrieved pair in the prefrontal representation (Fig 2B). The results were again accumulated to a threshold, where positive accumulation rate was higher than negative accumulation rate. If the pairs matched (targets), a positive response was issued, if not (re-paired foils), a negative response. Following a recent analysis [54], comparison was done word-by-word, after being fed through two so-called clean-up memories to remove noise from the representations [55,56]. We tentatively located the decision process in the posterior parietal cortex, though only found limited evidence for this assumption [9]. For this reason, we did not compare the model’s activation in this area to MEG data.

Finally, based on the outcome of the familiarity process or the decision process, a positive (index finger) or negative (middle finger) response was issued to the motor system. Based on the current experimental block either the right or the left hand was used. During the decision process, both fingers were already slightly activated, followed by the selected finger after the decision was made. The motor system consisted of a simple hierarchy of a higher-level motor buffer and low-level finger areas, which was linked to a region in the motor cortex (Fig 4E).

### Brain Model–Parameters

The development of the model consisted of two phases: structural development and parameter adjustment to match the human RT data. First, we build the structural connections of the brain model (Fig 2), implementing the ideas of the theoretical model shown in Fig 1. All modules were custom developed, except the basal-ganglia-thalamus system, for which we used the default Nengo implementation [35]. In this phase, we focused on whether the model reflected the qualitative effects in the behavioral data and in the MEG data. To this end we introduced changes to the basal-ganglia-thalamus system (short thalamic pulses) and the new Voja± learning rule, both described above. In addition, we implemented evidence accumulation processes for familiarity judgment, memory retrieval, and decision making, to increase the robustness and reliability of the model and match the time course of human cognitive processing. Finally, to better match the MEG data in occipital regions, we used adaptive LIF neurons [57] for part of the visual system, all other neurons are standard LIF neurons, with default Nengo parameters.

**Table 1:** Parameter values. Parameters that were adjusted for individual models.

Parameter	Min	Max	Mean
Positive drift rate familiarity	1	1.5	1.14
Negative drift rate familiarity	-0.13	-0.08	-0.10
Positive drift rate retrieval	0.004	0.007	0.0057
Comparison threshold	0.8	1	0.89
Negative drift rate comparison	-0.15	-0.03	-0.11

**Table 2:** Fit measures of the individually fitted models and the average model. Root-mean-square deviation (RMSD) is only reported for behavioral data, as we did not predict source activity quantitatively.

Data source	Individually fitted		Average model	
	*R* ^*2*^	*RMSD*	*R* ^*2*^	*RMSD*
Response time	0.97	64.02	0.96	74.52
Error rate	0.55	5.59	0.50	5.26
MEG - Visual	0.97		0.97	
MEG - Familiarity	0.83		0.83	
MEG - Retrieval	0.78		0.78	
MEG - Representation	0.27		0.34	
MEG - Right Motor	0.83		0.84	
MEG - Left Motor	0.69		0.69	
fMRI - Representation	0.60		0.59	

In the second phase, we performed parameter adjustment to match the human RT data more closely. Given the size and complexity of the model, automatic parameter fitting was not feasible (training the declarative memory system for one participant took about three days on an NVIDIA Titan X Pascal GPU, while simulating one participant doing the experiment took approximately 28 hours). Instead, we used a manual three-step procedure to match each simulated participant to the human RT data. This had our focus, because a good RT match is required for replicating the continuous MEG data.

First, for each simulated participant a different random seed was used. This seed determines the tuning curves of the neurons and other low-level neural properties, as these are drawn randomly from pre-defined distributions matching neural data [26], as well as the specific semantic pointers that are generated. In addition, each simulated participant received a different set of word pairs, reflecting the human experiment. Second, because of the neural properties slightly varying between participants due to different random seeds, the basal-ganglia complex could sometimes select an incorrect action. To ensure that each simulated participant went through the same actions, we made small adaptations to the actions such as adjusting the input strength of a cortical area or a utility threshold. Ideally, this would be learned through reinforcement learning [35], but that was outside the scope of the current model. These adaptations did not change the structure of the model or the fit to the data, but simply ensured that all simulated participants went through the same process. Third, to match the RT data, we adjusted the drift rates of the familiarity, retrieval, and comparison accumulators, as well as the comparison threshold (see Table 1; all other parameters were left at their default values). These rates were manually selected based on experience, with typically only a few runs per simulated participant given the time costs.

Using this process, we simulated 18 participants who each performed 1,120 trials of the associative recognition task. To test whether the brain model was generalizable, we also ran each simulated participant with the average of these parameters. Table 2 reports fit measures for the individually fitted models as well as the average model. Importantly, the model was not quantitatively fitted to the MEG or the fMRI data.

### Brain Model—Results

Fig 5 shows the RT data: fan-2 pairs are harder to learn than fan-1 pairs, resulting in higher RTs. This was matched by the model: representations of fan-2 pairs are more similar to each other than representations of fan-1 pairs, resulting in weaker memory encoding. Because the retrieval process ends when a retrieved representation is sufficiently clear, this process takes longer for fan-2 pairs, driving the main behavioral effect. In addition, re-paired foils have higher RTs than targets. This was simulated by using a higher positive than negative accumulation rate for the decision comparisons, reflecting that positive decisions are based on clear evidence in this task–that is, a word pair is retrieved from memory that is the same as the presented word pair–while negative decisions are due to an absence of evidence. In the latter case, it is possible that evidence still occurs (for example, due to noisy memory retrievals), so a lower drift rate is sensible (cf. words vs. non-words in lexical decision, e.g., [58]). Table 2 shows that this resulted in a convincing fit: both the individually fitted models as well as the average model had an R^2^ greater than .95, and an RMSD of at most 75 ms.

**Fig 6:**
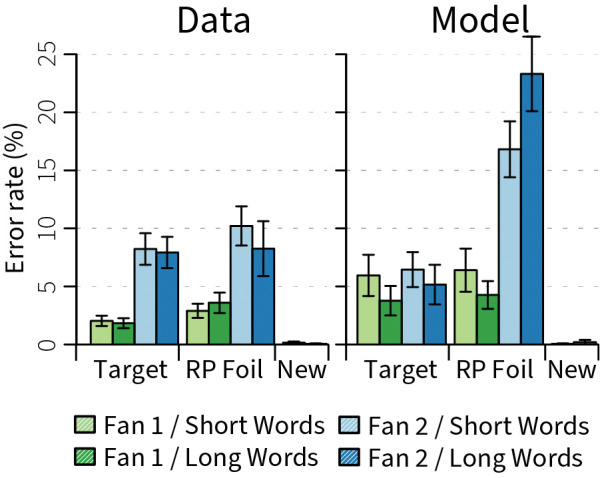
Error rates data and model fit. Error bars indicate standard errors of the mean. RP foil = re-paired foil.

The main effects in the error rates were also matched by the model (Fig 6): it made hardly any errors for new foils, more errors for fan-2 than fan-1 pairs, and more errors for re-paired foils than for targets. However, the model performed worse than the human participants on average, and made too many errors for fan-2 re-paired foils. This is reflected in the R^2^ value of .55 for the individually fitted models and .50 of the average models (Table 2). The errors originated mostly from incorrect retrievals: a wrong pair in case of targets, a pair resembling the encoded pair in the case of re-paired foils. In addition, sometimes the model did not manage to retrieve any meaningful information, leading to a time-out. These errors are probably due to the rather simple structure and limited number of neurons of our memory system, as compared to the human hippocampus [44].

Predicting MEG and fMRI data was done for correct trials only. For the MEG predictions, we assumed that source-localized MEG activity [20] reflects the sum of the neural spikes, resulting in qualitative predictions. For fMRI we predicted the BOLD response, which we assume is linearly related to the level of neurotransmitter usage in a region [59,60]. We therefore convolved the models’ neurotransmitter usage with the standard hemodynamic response function of the SPM software [61], as is common practice for predicting the BOLD response [14,62].

Fig 4A–4E shows that the model captured all main MEG effects, with hardly any differences between the models that were individually fitted to the RT data and the models using the average parameters (Table 1). The visual encoding process matched well to the data in occipital cortex, including the more pronounced activity for long words. However, in the data a second peak is visible around 300 ms, which is probably due to feedback to V1 (e.g., [63,64]), which was not included in the model. For both familiarity and retrieval, the model predicted a main effect of Fan, which remained to the end of the interval in Fig 4. However, the data show that this is not entirely correct: the differences disappeared at the end of the interval. This is probably due to the higher noise levels in the data, as well as to more variability in cognitive process and trial durations. With respect to the representation process, linked to the superior middle frontal region, Table 1 indicates a rather low fit with an R^2^ around .3. This is mainly due to differences in the timing of the effects between model and data: the data peak more clearly, and closer to the response than the model predictions. On first sight, this suggests that the response process in the model is too slow. However, this seems unlikely, given the strong match to the data in the motor cortex (Fig 4E). Apparently, in reality there is more overlap between representation and motor processes than currently present in the model, indicating that the model should have maintained the representation longer. This would lead to a later and more pronounced peak, as is visible in the data. On the other hand, even here the main effects are clearly present, including the crossover of fan-1 activity over fan-2 activity from the start to the middle of the interval.

Fig 4F shows the classic fMRI effect in associative recognition (reanalyzed data from [15]; see Materials and Methods): a stronger response to higher fan pairs than to lower fan pairs around the inferior frontal sulcus–opposite to the MEG effect in this region. We explain this seeming paradox by noting that the fMRI BOLD response is driven by the energy consumption of neurotransmitter usage across time [60], while MEG reflects direct neural activity. Since fan-2 trials have longer RTs, the model predicted a higher BOLD response for fan-2 pairs, even though it had a stronger direct neural response to fan-1 pairs. The difference between targets and foils is similarly due to the difference in RTs. Given that the setup of this study was somewhat different from the modeled experiment, and that we did not attempt to match these data, the resulting qualitative fit seems convincing.

## Discussion

Taken together, the current brain model shows how goal-directed, functional behavior could result from the low-level spiking activity of neurons. Although several brain models have been proposed in recent years [3,5], they were typically evaluated using local neural effects, qualitative behavioral patterns, or general neural phenomena [65]. Instead, here we evaluated the model on a cognitive task–associative recognition–using quantitative behavioral measures, continuous source-localized MEG data, and a classic fMRI effect. Based on the combined fit across these different measures, we argue that it provides a plausible hypothesis about how the human brain performs cognitive tasks. Importantly, the implications are not limited to associative recognition, as the model includes a variety of cognitive processes that are present in many, if not most, cognitive tasks.

In developing the model, we followed the approach recently advocated by Kriegeskorte and Douglas [6], who argued for the use of cognitive computational models to explain how cognition is implemented in the human brain, and testing these models on both behavioral and brain data. As they suggested, we used existing cognitive models [10,11,13,41] as the basis for the brain model, further informed by machine-learning analyses of neuroimaging data [9,12,39]. This gives the current brain model a strong foundation in cognitive theories, while being directly linked to neuroimaging data. However, some design decisions were made solely based to improve the fit to the data. For example, the new learning rule was introduced to explain the difference between fan 1 and fan 2 pairs in prefrontal cortex. While this resulted in a good fit for the current model, future experiments are required to evaluate the generality of this solution.

The top-down approach that was used to develop the model can be contrasted with the bottom-up approach typically used in computational neuroscience [4,6]. Whereas we focused on matching cognitive processes and matching composite brain measures, the brain models developed from a computational neuroscience perspective have been concerned with the biological plausibility and detail of the neurons. An early example is Markram’s Blue Brain Project, which simulated about one million neurons in tremendous biological detail [1,65]. Other broadly reported models are rat- and cat-scale brain models from Izhikevich and Modha [66,67]. While these models provided important insight into the complexity of the brain, for instance in the nonlinear properties of large ensembles of neurons (e.g., cortical columns [65]) and, for example, fit cortical dynamics, they were not able to simulate goal-directed behavior.

More recently, several models have been proposed that combine high biological plausibility with the performance of simple cognitive tasks (e.g., [68,69]). An interesting example is a large-scale model of the macaque cortex by Froudist-Walsh and colleagues [69]. The structure of this anatomically-constrained model consisted of 40 cortical areas, each of which was made up of a local circuit with pyramidal cells and three types of inhibitory neurons. The properties of these local circuits–long-distance connectivity, strength of excitation, and most importantly dopamine receptor density–were based on detailed analyses of neural data. While this model incorporates less biological detail than, for example, the work of Markram and colleagues [65], it was used to perform several working memory tasks, making it cognitively functional. As one result, the model contributed to a current discussion in cognitive and computational neuroscience on activity-silent vs. persistent-activity based working memory maintenance [e.g., 30,70–73], by showing that the type of maintenance depended on cortical dopamine levels.

When comparing it to the current model, several things stand out. On the one hand, the Froudist-Walsh (FW) model incorporated biological mechanisms to a much greater extent (with the one exception that the current model uses spiking neurons instead of population firing rates). On the other hand, while the FW model was used to simulate working memory maintenance, it did not provide responses, and was therefore not evaluated based on monkey behavior. Through the inclusion of the BG-based action-selection system in our model, it can perform more complex sequences of cognitive actions–and in principle multiple different tasks [5]–and therefore be evaluated both on behavior and neural data over time. Recently, we also proposed a model of activity-silent working memory using his approach [30]. This provides a nice contrast to the FW model: while our model showed the effect of using activity-silent maintenance on behavior across three tasks, the FW model explains the biology of activity-silent maintenance in the brain. The next step would be to combine both models, and explain under what circumstances the brain uses activity-silent memory, and how such a choice is implemented at the biological level.

The current model is most closely related to Spaun, the 2.5-million-neuron brain model proposed by Eliasmith and colleagues in 2012 [5]. Spaun was developed using a bottom-up computational neuroscience approach, while keeping cognitive functionality in mind. We applied the same modeling framework and incorporated mechanisms such as the basal-ganglia system. However, where Spaun could perform eight different tasks, the current model is only directly suitable for associative recognition. Conversely, the evaluation of Spaun was limited to local neural effects and qualitative behavioral effects, while the current model was compared quantitatively to behavioral data and qualitatively to source-localized MEG data across time. By constraining the model further, we made it more plausible from a cognitive perspective while profiting from the bottom-up approach that was used to develop Spaun. In the future, we aim to further develop the different modules of the current model, incorporating more low-level neuroscience constraints–as in the FW model ([69], see [74] for a Spaun-related example). At the same time, we plan to extend it to more different tasks.

Partly because of our focus on the evaluation with MEG data, the current brain model might seem highly localizational, with every module linked to a specific brain region (Fig 2). In contrast, current (cognitive) neuroscience highlights the importance of distributed processing across brain networks (e.g., [75,76]). However, even in such networks, one can typically identify a main region for each cognitive function. For example, we have previously identified main regions for working memory updates and declarative memory retrievals *within* the fronto-parietal control network [77]. The regions that were linked to the modules of the brain model are such “main regions”, which we view as processing hubs for particular cognitive functions. On the one hand, this means that while these are the most important regions for each function, the actual processing can be more distributed. On the other hand, it implies that we can use those main regions as indicators of cognitive functions, and therefore use them to evaluate the model (cf. [78,79]).

Using the source-localized MEG data led to several important advances in the brain model. First, to match the MEG data, we adjusted the existing basal ganglia action-selection system to use brief thalamic pulses [31,34,35] instead of continuous input to change the functional connectivity of the cortex. This is in line with current theories of EEG generation [36,37], which assume either a phasic burst of activity or a reset of the phase of the oscillations at the moment of a significant cognitive event–not an extended period of increased activation. Using a generative whole-brain biophysical oscillator model, we have recently shown that these effects can be caused by brief pulses from the thalamus, which nevertheless can lead to sustained periods of adjusted functional connectivity [39]. This adjusted basal ganglia-thalamus system resulted in a good fit to the MEG data, while the original system [35] led to longer periods of high neural activity that did not match the data. We hypothesize that the brain uses such brief thalamic pulses as it is more energy efficient than using continuous input, and leads to adequate performance.

Secondly, we devised a new unsupervised learning rule for the hippocampus. Unlike previous rules [46], strongly responding neurons are not only attracted to certain inputs, but weaker responding neurons are repelled. This results in very strong pattern separation, a sparse memory system that avoids catastrophic forgetting, and more ‘space’ for new memories, matching hippocampal data [44]. Without this new rule, fan-2 pairs consistently triggered more neural activity than fan-1 pairs, while the MEG data showed the opposite pattern. Somewhat surprisingly, even with this new rule the fMRI predictions still correctly showed a higher BOLD response for fan-2 pairs: even though fan-2 pairs triggered less moment-to-moment neural activity, the temporal filtering inherent to the BOLD response resulted in higher overall activity. This highlights the risk of using a delayed signal like the BOLD response without properly equating the duration of the underlying cognitive processes.

One might wonder how this rule could be implemented biologically. The direction of the changes to the connectivity strength depends on how strongly post-synaptic neurons respond to a certain input, so on the difference between the activity of the pre- and postsynaptic neurons–in effect it switches from Hebbian learning to anti-Hebbian learning when the activity is too low. This is similar to what is observed in STDP (e.g., [80]), although in that case it is based on the timing of spikes rather than the absolute level of spiking (see [81] for a potential connection between STDP and Hebbian learning). One place the spike rate might be available to the neuron would be calcium concentration (cf. [30,73]), though that tends to be more available at the axon than at the dendrites, which is where it would be needed here.

Finally, we used evidence accumulation mechanisms in several places in the model, most notably for familiarity judgement, retrieval judgement, and the decision-making process itself. This was necessary for two reasons: first of all, it led to more robust decision making as the evidence was accumulated over time. Secondly, without these accumulators the model made its decisions too fast, and its predictions neither fit the behavioral data, nor the MEG data. Such neural evidence accumulation systems have been proposed before, and shown to match human brain data [51–53].

In conclusion, using the MEG data led to the introduction of several biologically-plausible mechanisms in the current brain model, which resulted in robust behavior and a good match to the human data.

## Materials and methods

### Associative recognition task

The brain model performed an associative recognition task based on an EEG experiment [8] with 20 participants and an MEG [9] experiment with 18 participants. Both experiments used the same associative recognition task, except that the EEG experiment included a *new foil* probe type, and the MEG experiment included a *response hand* condition. The model performed the MEG experiment with the addition of new foil trials from the EEG experiment, because these new foil trials provided clear insight into the timing of the familiarity process. This synthetic ‘combined experiment’ consisted of the following conditions: *probe type* (target, re-paired foil, new foil), *associative fan* (words appeared in 1 or 2 pairs), *word length* (short or long words), and *response hand* (left or right). However, note that we do not have MEG data of *new foil* trials.

Both the EEG and MEG experiments consisted of two phases: a training phase and a test phase. In the training phase, participants studied 32 word pairs. Sixteen pairs consisted of fan-1 words and sixteen pairs consisted of fan-2 words. Half of the pairs in each of these categories was made up of short words (4–5 letters), and the other half of long words (7–8 letters). Word frequency was matched across all conditions. After an initial 5-second presentation of each word pair, participants performed four blocks of a cued recall task. On each trial, one word was presented, and the associated word (fan-1 pairs) or words (fan-2 pairs) needed to be entered (all separate words were presented, so associations were learned in two directions). If an error was made, this word was repeated at the end of the block. Thus, participants answered correctly four times to each word. In the MEG experiment, participants responded on average (correctly or incorrectly) 5 times to fan-1 words and 11 times to fan-2 words–indicating that fan-2 pairs are more difficult to learn than fan-1 pairs [9].

In the test phase of the MEG experiment, participants performed 14 blocks of the associative recognition task in a MEG scanner. Each block consisted of 32 target trials and 32 re-paired foils (re-arranged target pairs). In half of the blocks participants responded with their right hand, and in half of the blocks with their left hand (target = index finger, foil = middle finger). Each test trial started with the presentation of a fixation (400–600 ms), followed by the probe pair. After the participant responded, feedback was given for 1000 ms, followed by a blank intertrial interval of 500 ms (S1 Fig).

The model performed this MEG experiment, except that we added 16 new foil pairs (new words that were never repeated) to each block for the model, reflecting the EEG experiment. Figs 5 and 6 report target and re-paired-foil data from the MEG study [9] and new-foil data from the EEG study [8].

### MEG Analysis and ROI definition

To evaluate the brain model, we compared its neural activity to source-localized MEG activity in several regions of interest (ROIs) that were defined based on analyses of the original MEG study [9]. MEG data were recorded at 1 kHz with a 306-channel Elekta Neuromag (Elekta Oy) whole-head scanner [9]. After manually rejecting bad channels, a band-pass filter of .5–50 Hz was applied, and the data were down-sampled to 250 Hz. Eye blinks and saccades were removed through independent components analysis. The MEG sensor data were projected onto 5,124 sources (approximately 6 mm apart) on the cortical surface using cortically constrained minimum norm estimates (MNE) [20]. The 3D cortical surface models were based on the participants’ structural MRIs.

### fMRI analysis

To test whether the model can explain a classic associative recognition effect in the prefrontal cortex, we reanalyzed Experiment 2 of an fMRI study on associative recognition [15], as the authors did not report effects of re-paired foils in the original paper.

This experiment was similar to the MEG and EEG studies discussed above: participants first learned paired associates in a study phase, and were later tested in the fMRI scanner with an associative recognition task [15]. Here, fan ranged from 1 to 3; targets and re-paired foils were used as probes; and stimuli could be presented visually or aurally. Twelve participants performed 200 trials, equally divided over conditions. fMRI data were recorded on a Siemens 3T scanner, with a TR of 1.5 seconds.

Previously, we have reanalyzed this dataset in combination with a cognitive model in order to localize cognitive functions [77]. Here, we use the same preprocessed data. The functional images were realigned, coregistered with the structural images, normalized to Montreal Neurological Institute space, and smoothed with an 8-mm FWHM Gaussian kernel. Next, we excluded fan-3 trials, and selected data in a region consisting of 5 x 5 x 4 voxels, centered on MNI coordinates *x* = -43, *y* = 24, *z* = 25. This region has consistently been associated with memory retrieval and representation in the ACT-R cognitive architecture over the past two decades [14,16,17,62,79,82]. The % Blood-Oxygen-Level-Dependent (BOLD) signal change was calculated with respect to the first scan of each trial. We collapsed over the visual and aural conditions.

### Brain model

Fig 2A shows the main modules of the brain model and Fig 2B shows the detailed functional architecture. General properties of different parts of the model are listed in S2 Table. All connections were pre-calculated, except for connections in the memory system. Below we discuss details of model components that are not in the main text.

### Neural vector representations

Nengo uses vector representations, which can be encoded and decoded by populations of spiking neurons [26]. Here we used default LIF neurons. Each neuron *i* has an encoding vector *e*_*i*_, which indicates the preferred direction of the neuron, that is, to which direction in vector space the neuron will react most. In addition, each neuron has a gain *α*_*i*_, and a background current $J_{i}^{bias}$. Together with the input vector *x*, this determines the input current for a neuron:

$$J_{i}(t)=\alpha_{i}e_{i}∙x(t)+J_{i}^{bias}$$ (1)

where ⋅ indicates a dot product. Next, this input current is converted into a spike train *a*_*i*_ through the use of the LIF non-linearity *G*_*i*_:

$$a_{i}(t)=G_{i}[J_{i}(t)]$$ (2)

In the case of the LIF non-linearity, the subthreshold voltage is described by

$$\dot{V}(t)=-\frac{1}{\tau_{RC}}(V(t)-J(x)R)$$ (3)

where *V* is the membrane voltage, *J(t)* the input current, *R* the passive membrane resistance, and *τ*_*RC*_ the membrane time constant. Every time *V* exceeds a threshold, the neuron emits a spike and is reset for time period *τ*_*ref*_, the refractory time constant. From a set of spike trains, vectors can be decoded:

$$y(t)=\sum_{i}d_{i}(a_{i}*h)(t)$$ (4)

First, each spike train is convolved with a low-pass filter *h(t)* representing the post-synaptic current, which is then multiplied by decoding vector *d*_*i*_.

The product of the decoders and encoders defines the weight matrix between neural populations:

$$\omega_{ij}=d_{i}e_{j}$$ (5)

By default, these connection weights are pre-calculated by Nengo to approximate a given function, but they can also be learned and adapted through various learning rules [28–30]. Finally, combining the above gives the complete input of a postsynaptic neuron *j*:

$$J_{j}(t)=\sum_{i}\alpha_{j}\omega_{ij}(a_{i}*h)(t)+J_{j}^{bias}$$ (6)

In other words, the general approach is to define small (single-hidden-layer) neural networks composed of spiking neurons, each one trained to perform a separate function, and these components are combined together to create the full model. For example, in the “Representation” part of Fig 2, we train a simple feed-forward neural network with a single hidden layer of LIF neurons where the inputs and outputs are randomly chosen 512-dimensional vectors (and no non-linearities are used at the input or output). Since we want this network to store information, we train it to approximate the identity function *y* = *f*(*x*) = *x*. We then take the output from this network and connect it back to its own input. Importantly, since there are no non-linearities at the input and output, we can combine the connection weights *e* (from the input to the hidden layer; Eq 1) and *d* (from the hidden layer to the output; Eq 4) into a single weight matrix *w* (Eq 5). This is the recurrent weight matrix between the hidden layer neurons that will cause those neurons to store vectors over time.

All other connections in our model are generated in a similar way, training networks to compute the desired function, and combining the output weights *d* with the input weights *e* to generate the connection weight matrices that go from one network’s layer of LIF neurons to the next network’s layer of LIF neurons. The final model, then, only contains LIF neurons and connection weights between them. These neurons and connection weights implicitly encode the input (*x*) and output (*y*) vectors that are used to build the model. For simplicity, when training these original networks, we randomly generate the input weights *e* and use least-squares minimization (ridge regression) to find the output weights *d*.

Given that the connection weights are purely based on the vector representations, this means that neural activity itself is not transferred directly between populations, but only the information represented by the vectors.

### Memory system implementation

Fig 3 shows our simplified implementation of Norman and O’Reilly’s CLS framework [2,42]. Both familiarity memory and declarative memory consist of a main population and an output layer, and receive input from other parts of the model. The main populations consist of 30k LIF neurons each. The tuning curves of these neurons have relatively high intercepts, making the neurons selective of their input and leading to sparse representations. The output layers consist of 50 neurons per dimension, which results in ~26k neurons, given that we used 512 dimensional semantic pointers. The locally-supervised Prescribed Error Sensitivity (PES) learning rule [43] was applied to the connection between the main populations and the output layers to learn to represent the training input:

$$\Delta d_{i}=\kappa ra_{i}$$ (7)

where *d* are the decoders of the main population, κ the learning rate, *r* the error, and *a*_*i*_ the activity of neuron *i*.

As explained in the main text, we added Hebbian learning to the incoming connections of the declarative memory system, using a new learning rule: Voja±. Eq 8 shows the Voja± rule for the preferred direction vector *e*_*i*_ of neuron *i*:

$$\Delta e_{i}=\left\{ \begin{array}{c} 0,a_{i}=0\\ \frac{\eta a_{i}(x-e_{i})}{a_{i}-\sigma },a_{i}>0 \end{array}\right.$$ (8)

which is updated depending on the difference with input vector *x*, its neural activity *a*_*i*_, the learning rate *η*, and the threshold *σ*. The learning rule causes the preferred direction vectors of neurons with *a* > *σ* to be attracted to the input vector, while the preferred direction vectors of neurons with *a* < *σ* are repelled. The closer the activity is to the threshold, the stronger the effect. These preferred direction vectors are encoded in the connection weights between the input and the neural population.

To bind the elements of an activated pattern together, we used an all-to-all recurrent connection on the main population of the declarative memory system. The weights on this connection were learned using unsupervised Hebbian BCM learning [48]:

$$\Delta \omega_{ij}=\kappa a_{i}a_{j}(a_{j}-\theta )$$ (9)

where *a*_*i*_ and *a*_*j*_ are the activity of the pre- and post-synaptic neuron, respectively.

Unlike Norman and O’Reilly’s combined CLF model [2], we used two fully separate memory systems in order to keep development and training manageable, even though in the brain these systems are most likely partly integrated. The memory systems were trained before we ran the main simulation. They were presented with the fan-1 and fan-2 words (familiarity) and pairs (declarative memory) at the same rates as the human participants. Presentation duration for fan-1 items was 1 second and for fan-2 items 500 ms, to reflect that fan-2 items had to be learned in the presence of two other words, and fan-1 items in the presence of one other word.

### Action selection implementation

To implement action selection, we needed two core components: a method for determining which action to perform at any given time, and a method for actually causing that chosen action to be performed. For this, we followed the standard idea of a cortex-basal ganglia-thalamus loop. As the first component, the cortex stores the current informational state. The connections between the cortex and the inputs to the basal ganglia (striatum and subthalamic nucleus) compute how well this current state matches the conditions for each pre-determined action, and the basal ganglia selects the highest-ranked action. For the second component, the output from the basal ganglia to the thalamus (via the globus pallidus internus) inhibits all actions except for the one chosen. Finally, the thalamus controls the flow of information between different cortical regions–based on the chosen action–through gating mechanisms.

The computation of how well-suited each action is for the current situation (the connections from cortex into the basal ganglia) is done by expressing these conditions as a function on vectors (*y* = *f*(*x*), where *y* is a scalar indicating the current utility of an action and *x* is the current state of the cortex), and using the techniques in the previous “Neural vector representations” section to find the correct connection weights that approximate that function. Ideally, at any given time one of these values will be large and the others will be small, indicating which action should take place.

For the basal ganglia portion of the model, we started with the mathematical (non-spiking) model of the basal ganglia as an action selection system proposed by Gurney and colleagues [83] and then used small (50-LIF-neuron) networks to approximate each of the internal calculations needed in their model. We also constrained the connection weights to be excitatory or inhibitory in accordance with the known neurochemistry of the basal ganglia. This is the identical model as has been used to model the Tower of Hanoi task [84], learning a Bandit task [35], and in Spaun [5]. The overall system can be thought of as a winner-take-all system that has been unrolled in order to have better temporal dynamics and scales up to large numbers of actions.

However, for this model we adjusted how this basal ganglia model was used, in order to better match recent theories on EEG generation [12,36–38]. Instead of using continuous input from the thalamus to change the functional connectivity of the cortex, here we used brief pulses that caused sustained periods of adjusted effective connectivity [39]. In the current model we implemented this by using two action rules for each cognitive action: one action that briefly changes the cortical connectivity, and one action that maintains processing until another pre-defined striatal state is reached, which will cause another action rule to be executed. We only applied this system to longer actions (familiarity judgement and memory retrieval), as it does not make a difference for brief actions.

### Visual encoding

The model encoded the word stimuli by parsing images (90x14 pixels) of the words with 2,000 adaptive LIF neurons that use 9x9 Gabor filters as tuning curves [26,85]. The images activated 384-dimensional representations of the words, which in turn activated 512-dimensional semantic pointers that represent the lexical concepts of the words in a concept population. To maintain the encoded word pair over the course of the trial, these concepts were stored in a visual buffer with a strong recurrent connection. As word order is relevant in associative recognition tasks, the pair is represented as ITEM1 ⊗ *word1* + ITEM2 ⊗ *word2*, where ITEM1 and ITEM2 are fixed semantic pointers, *word1* and *word2* are the activated word concepts, and ⊗ is the circular convolution operator to bind them together [26,27].

### Declarative memory and representation

During the retrieval process, the output pair was stored in the representation population, which maintained the information through a recurrent connection. As explained above, in the Nengo framework only information is transferred between neural populations, not the neural activity itself. Here, we additionally simulated the apparent carry-over of neural activity from the hippocampus to the prefrontal cortex [9].

### Control

As explained above, action selection was performed by the basal ganglia. Although the state of the cortex generally indicated the next step in the process, we additionally included a number of control states, as is common practice in higher-level computational models of cognition [14,29]. To be precise, we used states for representing the current step in the process (encoding, retrieval, etc.), the current response hand (left, right), and the currently attended word (first, second). Finally, in addition to the gated connections controlled by the basal ganglia, we also used several structural connections: between the visual buffer and the familiarity and declarative memory systems, and between declarative memory and the prefrontal representation. These connections provided continuous transfer of information between those systems, which could be strengthened by the basal ganglia-thalamus system.

### Model predictions temporal cortex

It was assumed that the middle temporal cortex was most indicative of a familiarity process, and the superior temporal cortex of a retrieval process [9]. However, source-localized activity in both regions reflects a mixture of the underlying brain regions: the perirhinal cortex, associated with familiarity, and the hippocampus, associated with retrieval. Model fits for these regions in Fig 4 are therefore mixtures of the familiarity and memory populations’ activity, where the first peak is due to the familiarity process, and the second peak to retrieval. In case of the familiarity predictions, the familiarity activity was weighted 60% and the declarative memory activity 40%, while in the case of the retrieval predictions, the declarative memory activity was weighted 55%, and the familiarity activity 45%. These weights were based on visual inspection of the results. The pure familiarity and declarative memory predictions are shown in S2 Fig.

## Supporting information

S1 Fig Trial structure. ITI = inter-trial interval.
(PDF)

S2 Fig **Pure activity of the (A) familiarity and (B) retrieval systems.** Because the hippocampus and perirhinal cortex are adjacent, both regions contribute to ‘familiarity’ and ‘retrieval’ data. Model fits in Fig 3 are therefore mixtures of the familiarity and memory populations; here the pure activity is shown. Foil = re-paired foil.
(PDF)

S1 Table Origin and properties of MEG ROIs. Origin lists the contrast from the non-parametric permutation tests and cortical regions (Desikan-Killiany atlas [23]) as identified by the classification analysis in the original paper [9] that were intersected to create the ROI. Properties lists the size and center-of-mass MNI coordinates of the ROI. L = left, R = right. The *word length* contrast for Visual Encoding was uncorrected for multiple comparisons following the original analysis [9].
(PDF)

S2 Table Modules of the brain model. Brain regions in italics were identified based on the literature. Neuron counts also include gates that are required for basal ganglia functioning.
(PDF)
