# Supplementary figures and images for: A whole-task brain model of associative recognition that accounts for human behavior and neuroimaging data
Source: PLoS Comput Biol. 2023 Sep 8;19(9):e1011427. doi: 10.1371/journal.pcbi.1011427 (PMC10511112; doi:10.1371/journal.pcbi.1011427)

Fixation

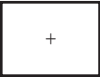

400-  
600 ms

Probe

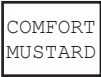

Until  
response

Feedback

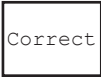

1000 ms

ITI

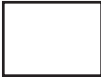

500 ms

Supplement: S1 Fig — ITI = inter-trial interval. (PDF) [file pcbi.1011427.s001.pdf]

# Model

**A****Familiarity**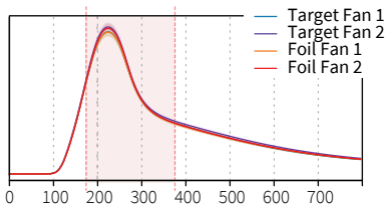**B****Retrieval**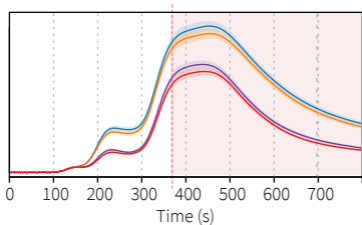

Supplement: S2 Fig — Pure activity of the (A) familiarity and (B) retrieval systems. Because the hippocampus and perirhinal cortex are adjacent, both regions contribute to ‘familiarity’ and ‘retrieval’ data. Model fits in Fig 3 are therefore mixtures of the familiarity and memory populations; here the pure activity is shown. Foil = re-paired foil. (PDF) [file pcbi.1011427.s002.pdf]
