# Supplementary material for: A whole-task brain model of associative recognition that accounts for human behavior and neuroimaging data
Source: PLoS Comput Biol. 2023 Sep 8;19(9):e1011427. doi: 10.1371/journal.pcbi.1011427 (PMC10511112; doi:10.1371/journal.pcbi.1011427)
Supplement: S1 Table — Origin lists the contrast from the non-parametric permutation tests and cortical regions (Desikan-Killiany atlas [23]) as identified by the classification analysis in the original paper [9] that were intersected to create the ROI. Properties lists the size and center-of-mass MNI coordinates of the ROI. L = left, R = right. The word length contrast for Visual Encoding was uncorrected for multiple comparisons following the original analysis [9]. (PDF) [file pcbi.1011427.s003.pdf]

S1 Table. Origin and properties of MEG ROIs. Origin lists the contrast from the non-parametric permutation tests and cortical regions (Desikan-Killiany atlas [1]) as identified by the classification analysis in the original paper [2] that were intersected to create the ROI. Properties lists the size and center-of-mass MNI coordinates of the ROI. L = left, R = right. The *word length* contrast for Visual Encoding was uncorrected for multiple comparisons following the original analysis.

**Cognitive Mechanism**	**Origin**		**Properties**	
	Contrast	*Cortical Regions*	*Size*	*Coordinates*
Visual Encoding	Word length (stimulus locked)	L Lateral Occipital & Lingual<br>R Lateral Occipital & Lingual	142<br>144	-25, -79, -2<br>25, -78, -1
Familiarity	Fan (stimulus locked)	L Middle Temporal	40	-55, -18, -19
Retrieval	Fan (response locked)	L Superior Temporal, Insula, Transverse Temporal	134	-47, -17, 1
Representation	Fan (response locked)	L Caudal Middle Frontal, Superior Frontal, Rostral Middle Frontal	130	-22, 17, 43
Left Motor	Response hand (response locked)	R Precentral	107	39, -9, 42
Right Motor	Response hand (response locked)	L Precentral	102	-38, -12, 45

References

1. Desikan RS, Segonne F, Fischl B, Quinn BT, Dickerson BC, Blacker D, et al. An automated labeling system for subdividing the human cerebral cortex on MRI scans into gyral based regions of interest. NeuroImage. 2006 Jul 1;31(3):968–80.

2. Borst JP, Ghuman AS, Anderson JR. Tracking cognitive processing stages with MEG: A spatio-temporal model of associative recognition in the brain. NeuroImage. 2016;141:416–30.
