# Supplementary material for: A whole-task brain model of associative recognition that accounts for human behavior and neuroimaging data
Source: PLoS Comput Biol. 2023 Sep 8;19(9):e1011427. doi: 10.1371/journal.pcbi.1011427 (PMC10511112; doi:10.1371/journal.pcbi.1011427)
Supplement: S2 Table — Brain regions in italics were identified based on the literature. Neuron counts also include gates that are required for basal ganglia functioning. (PDF) [file pcbi.1011427.s004.pdf]

**S2 Table. Modules of the brain model.** Brain regions in italics were identified based on the literature. Neuron counts also include gates that are required for basal ganglia functioning.

| Module | Neuron Count | Brain region |
|---|---|---|
| Visual encoding | 4,000 | Lateral occipital cortex |
| Visual buffer | 76,880 | Not identified. |
| Lexical concepts | 5,050 | *Dorsal temporal cortex [1]* |
| Familiarity memory | 225,150* | Perirhinal cortex |
| Declarative memory | 74,590 | Hippocampus |
| Representation | 64,040 | Dorsolateral prefrontal cortex |
| Decision | 189,680 | *Posterior parietal [1,14]* |
| Motor | 96,650 | Precentral |
| Action selection | 8,800 | Basal ganglia, thalamus |
| Control states | 64,500 | *Anterior cingulate [14]* |

*Note that Familiarity Memory neuron count includes a comparison population to compare input and output of the familiarity system.

**References**


1. Borst JP, Ghuman AS, Anderson JR. Tracking cognitive processing stages with MEG: A spatio-temporal model of associative recognition in the brain. NeuroImage. 2016;141:416–30.

2. Anderson JR. How Can the Human Mind Occur in the Physical Universe? New York: Oxford University Press; 2007.
